# Sequential Reactions of Acetylene with the Benzonitrile Radical Cation: New Insights into Structures and Rate Coefficients of the Covalent Ion Products

**DOI:** 10.1021/acs.jpclett.4c02496

**Published:** 2024-10-29

**Authors:** Paige Sutton, John Saunier, Ka Un Lao, M. Samy El-Shall

**Affiliations:** Department of Chemistry, Virginia Commonwealth University, Richmond, Virginia 23284-2006, United States

## Abstract

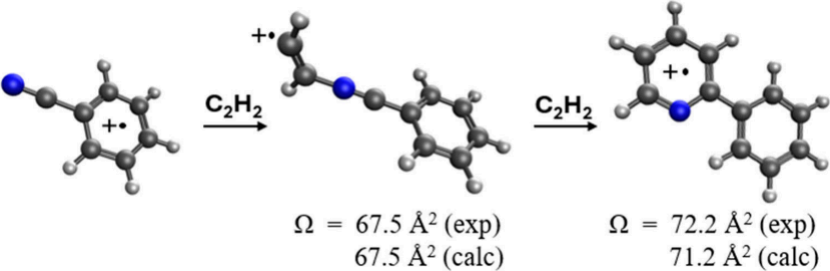

Benzonitrile radical cations generated in ionizing environments such as solar nebulae and interstellar clouds can react with neutral molecules such as acetylene to form a variety of nitrogen-containing complex organics. Herein, we present results from mass-selected ion mobility experiments and coupled-cluster and DFT calculations for the sequential reactions of acetylene with the benzonitrile radical cation (C_7_NH_5_^+•^). The results reveal the formation of two covalently bonded adduct ions C_9_NH_7_^+•^ and C_11_NH_9_^+•^ with individual rate coefficients of 2.1(±0.4) × 10^–11^ cm^3^ s^–1^ and 1.1(±0.9) × 10^–11^ cm^3^ s^–1^, respectively measured at 334.5 K. The direct addition of acetylene onto the N atom of the benzonitrile cation results in the formation of a N-acetylene-benzonitrile^+•^ radical cation with a calculated collision cross-section of 67.5 Å^2^ in perfect agreement with the measured cross-section of 67.5 Å^2^ of the C_9_NH_7_^+•^ adduct. The measured collision cross-section of the second covalent adduct C_11_NH_9_^+•^ (72.2 Å^2^) is also in excellent agreement with the calculated cross-section (71.2 Å^2^) of the lowest energy isomer of the C_11_NH_9_^+•^ ion corresponding to the 2-phenylpyridine structure. The formation of the bicyclic 2-phenylpyridine radical cation is explained by the rapid conversion of the classical radical cation C_11_NH_9_^+•^ into a distonic ion structure that can efficiently cyclize in an exothermic transformation to form the 2-phenylpyridine radical cation. This intriguing mechanism could explain the formation of N-containing complex organics in different regions of outer space. The current results are expected to have direct implications for the search for nitrogen-containing complex organics in space.

Gas phase ion–molecule reactions provide efficient mechanisms for the formation of complex organics including polycyclic aromatic hydrocarbons (PAHs) and polycyclic aromatic nitrogen heterocyclics (PANHs) found in solar nebula and interstellar clouds as well as in flames and combustion processes.^1−7^ Understanding the formation and growth mechanisms requires a detailed knowledge of the sequences of ion–molecule reactions leading to the formation of molecular cyclic ions such as benzene, pyridine, pyrimidine, and benzonitrile and the mechanisms by which these ions can react with neutral molecules such as acetylene to form PAHs and PANHs.

Because acetylene is regarded as the smallest organic molecule that can participate in polymerization reactions, the addition reactions of acetylene onto ionized aromatics have been considered as plausible efficient mechanisms for the formation of functionalized aromatics found in meteorites, interstellar medium, and combustion products.^8−22^ For example, the sequential reactions of acetylene with the benzene radical cation have been investigated using the mass-selected ion mobility technique.^13,15,16,20^ These studies have shown that the benzene radical cation can autocatalyze the conversion of three associated acetylene molecules into a benzene molecule at low temperatures such as 120 K, while at high temperatures such as 600–650 K, the benzene cation can react with two acetylene molecules to form naphthalene-type covalent products.^13,16,20^ In fact, the benzene radical cation remains largely unreactive with acetylene at 300 K, but when the temperature is increased to 600–650 K, the energy barrier associated with the C–C covalent bond formation can be overcome, leading to two sequential additions of C_2_H_2_ molecules onto the C_6_H_6_^+•^ with the elimination of a H atom to produce a protonated naphthalene species (C_10_H_9_^+^).^16,20^ Interestingly, the same type of species (C_10_H_9_^+^) can be formed by the sequential addition of two acetylene molecules onto the phenyl cation (C_6_H_5_^+^) near room temperature (307 K), suggesting that the barrier to C–C bond formation on the cyclic ion can be significantly reduced by the elimination of a hydrogen atom from the benzene ring.^17,20^ Alternatively, this reaction barrier can be overcome through the incorporation of one or two nitrogen atoms into the cyclic ion as with the pyridine (C_5_H_5_N^+•^) or the pyrimidine (C_4_H_4_N_2_^+•^) radical cations, respectively.^18^ In fact, the acetylene reaction with C_5_H_5_N^+•^ results in the formation of a covalent C–N bond with a reaction efficiency of about 10%, while the reaction with C_4_H_4_N_2_^+•^ proceeds at nearly the Langevin capture rate (1.5 × 10^–9^ cm^3^ s^–1^), thus providing nearly 100% efficiency for the formation of a C–N covalent bond at room temperature.^18^ The sequential reactions of two acetylene molecules with C_5_H_5_N^+•^ and C_4_H_4_N_2_^+•^ led to the elimination of a hydrogen atom and the formation of PANH cations such as the quinolizinium cation (C_9_H_8_N^+^) and the pyrid[1,2-α]pyrimidinium cation (C_8_H_7_N_2_^+^), respectively.^18^

The recent discovery of benzonitrile as the first aromatic molecule observed in the interstellar medium,^23^ and the subsequent detection of the PAHs 1- and 2-cyanonaphthalene in the cold molecular cloud Taurus Molecular Cloud 1 (TMC-1),^24^ have increased interest in the sequential acetylene reactions with the benzonitrile radical cation since these reactions can act as potential precursors for the nitrogen-containing PAHs in space which could provide a possible chemical link to the carriers of the unidentified infrared bands.^25−29^ Our previous study of the reactions of acetylene with the benzonitrile radical cation established the formation of two covalently bonded adducts at higher pressures of acetylene but lacked experimental structural information and measurements of individual rate coefficients of the first and second reaction products.^20^ Here, we investigate the acetylene sequential reactions with the benzonitrile radical cation at a low number density of acetylene to determine the individual rate coefficients for the formation of the first and second covalent adducts. Rate coefficient measurements revealed reaction efficiencies of 1.4 % and 0.7 % at 334.5 K for the formation of the first and second covalent adducts, respectively. Structural information is provided by measuring the collision cross-section of the first and second covalent adducts in helium using the mass-selected ion mobility technique.^11,19^ To gain deeper insight into the reaction products, the experimental results are complemented by simulations using the coupled-cluster with singles, doubles, and perturbative triples gold-standard method [CCSD(T)], along with density functional theory (DFT). The combined mobility measurements and structural calculations revealed the formation of an N–C bond by direct addition of the first acetylene molecule onto the N atom of the benzonitrile radical cation followed by the formation of the terminated product 2-phenylpyridine upon the addition of the second acetylene molecule. The formation of the bicyclic product 2-phenylpyridine at 334 K is intriguing and suggests a novel reaction pathway involving the formation of a distonic ion that could trigger an exothermic rearrangement from an open chain structure of the second acetylene adduct to the 2-phenylpyridine structure. The present results are expected to motivate the search for benzonitrile-acetylene covalent adducts in the cold-core TMC-1, which is known to involve a rich chemistry dominated by reactions of unsaturated molecules containing nitrile (R–C≡N) groups.^23−28^

Figure 1 displays the mass spectra obtained following the injection of the benzonitrile radical cation (B^+•^, C_6_H_5_CN, *m*/*z* 103) into the drift cell containing 2.5–3.5 Torr helium and variable amounts of acetylene (A, C_2_H_2_) at different temperatures. Figure 1 (a) shows no fragmentation of the benzonitrile radical cation, which confirms the lack of high ionization and injection energies that could result in the dissociation of the benzonitrile molecular ion. At a very low pressure of C_2_H_2_ (0.005 Torr), the first [B^+•^(A), C_9_NH_7_^+•^, *m*/*z* 129] and the second [B^+•^(A)_2_, C_11_NH_9_^+•^, *m*/*z* 155] adduct ions are readily formed at room temperature as shown in Figure 1(b). Increasing the temperature of the drift cell results in a small decrease in the ion intensity of the first and the second adduct and a corresponding increase in the intensity of the benzonitrile ion, as shown in Figure 1(c) to (g). However, at the highest temperature used (532 K), the adducts B^+•^(A) and B^+•^(A)_2_ still maintain most of their original ion intensities, indicating that the adducts contain a mixture of noncovalent and covalent ions. As the temperature increases, the weakly bonded noncovalent ions dissociate by the loss of acetylene, thus resulting in increasing the intensity of the benzonitrile ion. The ion intensities of the two adducts observed at temperatures as high as 532 K (Figure 1(g)) represent mostly covalently bonded ions that are thermally stable at these temperatures. This result is consistent with the thermal stability of the B^+•^(A) and B^+•^(A)_2_ adducts formed by the reactions of acetylene with the benzonitrile radical cation at higher pressures of acetylene (0.12 Torr) and at temperatures as high as 593 K.^20^

**Figure 1:**
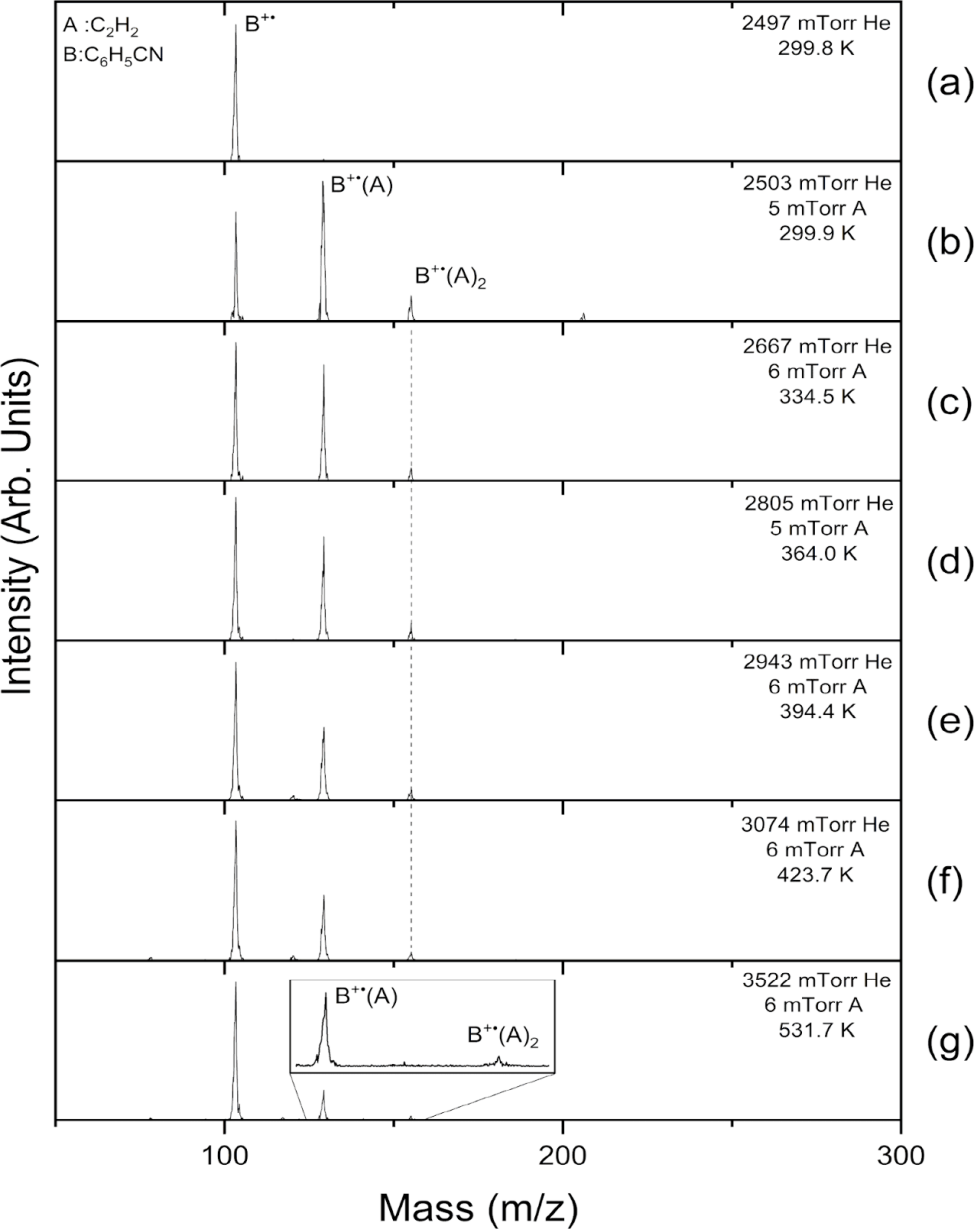
Mass spectra obtained following the injection (15 eV) of the benzonitrile radical cation (B^+•^, C_6_H_5_CN, *m*/*z* 103) into (a) a room temperature drift cell with 2.5 Torr of He and no acetylene (A, C_2_H_2_) and (b–g) with a starting acetylene pressure of 0.005 Torr from 300 K up to 532 K.

The product distribution of adducts B^+•^(A) and B^+•^(A)_2_ depends strongly on the pressure of acetylene in the drift cell. As shown in Figure S2 (Supporting Information), with a small increase in the partial pressure of acetylene from 0.008 to 0.012 Torr, the intensity of the benzonitrile ion decreases, and the intensities of the B^+•^(A) and B^+•^(A)_2_ adduct ions increase. This result is also consistent with the previously obtained result where the benzonitrile ion reacted almost completely at 0.10 Torr of acetylene to produce the first and second adducts (B^+•^(A) and B^+•^(A)_2_) as the two major products at 300 K.^20^ However, when the pressure of acetylene was further increased (0.20–0.5 Torr), the second adduct B^+•^(A)_2_ (C_11_NH_9_^+•^, *m*/*z* 155) became the most abundant ion, and only a very small amount of the third adduct B^+•^(A)_3_ (C_13_NH_11_^+•^, *m*/*z* 181) was observed. This indicates that a terminated product that has a very low reactivity toward the reaction with the third acetylene molecule is formed by the addition of two acetylene molecules onto the benzonitrile radical cation. A plausible structure of the terminated product C_11_NH_9_^+•^ could be a bicyclic ion that has no reactive carbon sites involving H loss for the addition of the third acetylene molecule. The structure of the B^+•^(A)_2_ adduct ion will be investigated by ion mobility and DFT calculations as described below.

Arrival Time Distributions (ATDs) of the product ions at 334.5 K are determined by varying the voltage gradient across the drift cell at two different partial pressures of acetylene (1.2 × 10^–3^ and 3.6 × 10^–3^ Torr) as shown in Figure 2(a) and (b), respectively. The residence time of the ions inside the drift cell increases by decreasing the voltage gradient across the drift cell, and as a result, the product yields into the two adducts C_9_NH_7_^+•^ (*m*/*z* 129) and C_11_NH_9_^+•^ (*m*/*z* 155) increase as shown in Figure 2(c) and (d), respectively. The formation of covalent ions as suggested by the concentration and temperature dependences of the product ions is consistent with the irreversible nature of the sequential acetylene reactions. The formation of noncovalent cluster ions involves reversible additions of neutral molecules into the parent ion, which, under equilibrium conditions, typically results in identical ATDs of the reactant and product ions since they are coupled by reversible association–dissociation reactions as they travel across the drift cell.^12,15,16,30^

**Figure 2:**
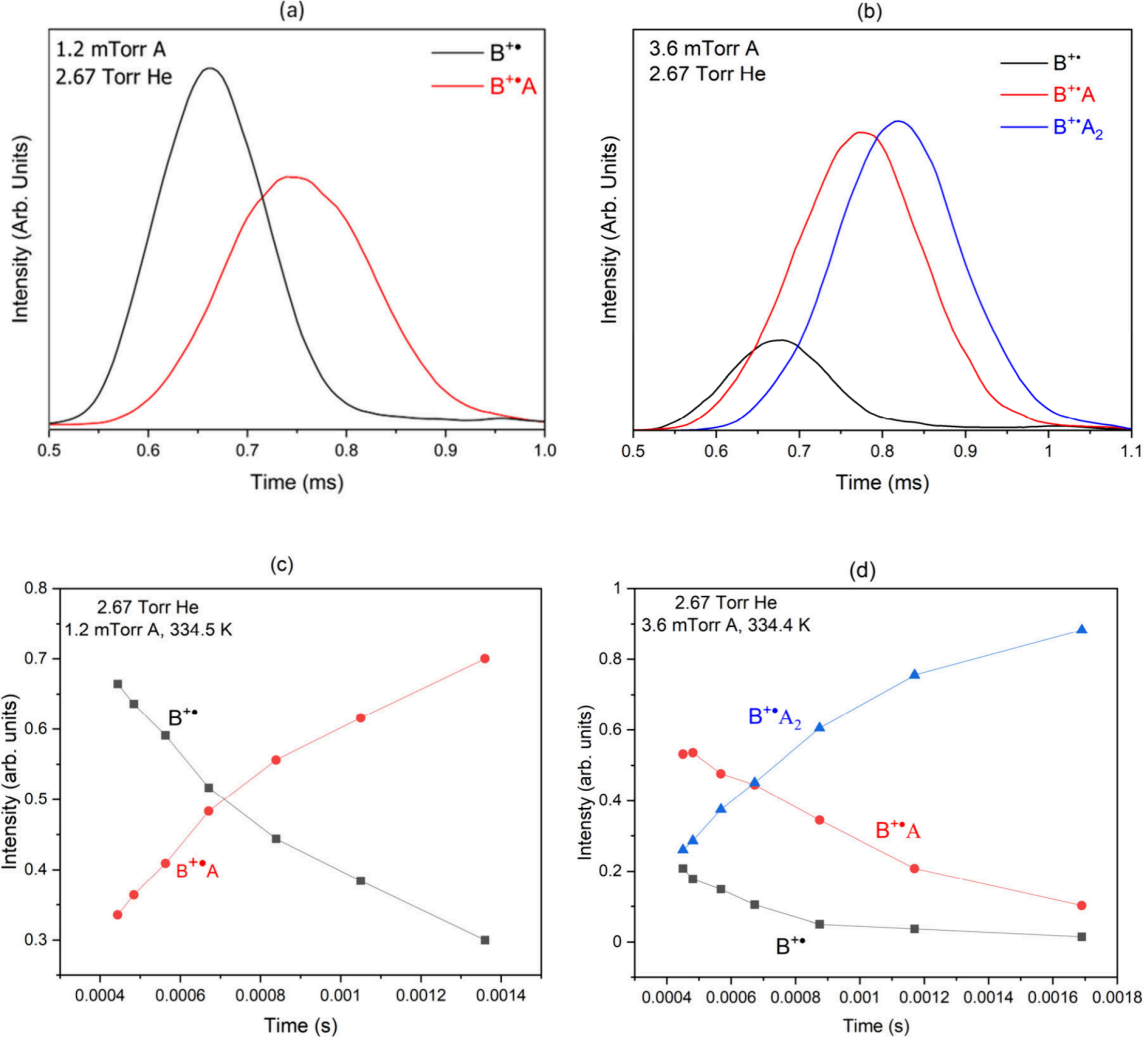
Arrival time distributions (a–b) of the reactant and product ions and the time profiles (c–d) for the reaction of the benzonitrile radical cation (B^+•^, black line) with acetylene (A) resulting in the formation of the first (c) (B^+•^A, C_9_NH_7_^+•^, red line) and the second (d) (B^+•^A_2_, C_11_NH_9_^+•^, blue line) adducts at a cell voltage of 12 V and drift cell length of 5 cm.

The second order rate coefficient for the formation of the first adduct (B^+•^A, C_9_NH_7_^+•^) is measured following the injection of the reactant ion (B^+•^, C_7_NH_5_^+•^) into the drift cell containing a very low pressure of acetylene (A) (1.2 × 10^–3^ Torr) in the presence of 2.67 Torr of helium as a third body buffer gas at 334.5 K as shown in Figure 2(c). As shown in Figure 2(a) and (c), there is no contribution from the second adduct to the total ion intensity observed during the reaction time. Pseudo-first-order rate constants are calculated from the equation: ln *I*/Σ*I* = −*kt*, where *I* is the integrated intensity of the ATD peak of the reactant ion (B^+•^, C_7_NH_5_^+•^), and Σ*I* is the sum of the integrated intensities obtained from the areas of the ATDs of the reactant and the product ion peaks (B^+•^ + B^+•^A), and *t* is the mean drift time measured at the center of the ATD peak corresponding to the reactant ion.^20^ The plots of ln *I*/Σ*I* vs *t* and the calculated slopes (pseudo first-order rate coefficients *k*_1_) are shown in Figure S3 (Supporting Information). The second-order rate constants *k*_2_ are obtained from the equation: *k*_2_ = *k*_1_/[*N*], where *N* is the number density of acetylene in the drift cell (3.6× 10^13^ – 1 × 10^14^ cm^–3^). The second order rate coefficient for the formation of the second adduct (B^+•^A_2_, C_11_NH_9_^+•^) is measured in the presence of relatively low pressure of acetylene (A), (3.6 × 10^–3^ Torr) in the presence of 2.67 Torr helium at 334.4 K as shown in Figure 2(d). Under these conditions, the ion intensities of the reactant B^+•^A (C_9_NH_7_^+•^) and product B^+•^A_2_ (C_11_NH_9_^+•^) ions observed during the reaction time represent more than 80% of the total ion intensity as shown in Figure 2(b) and (d).

The rate coefficients for the formation of the first and second adducts at 334.5 K are determined as 2.1(±0.4) × 10^–11^ cm^3^ s^–1^ and 1.1(±0.9) × 10^–11^ cm^3^ s^–1^, respectively as shown in Table 1. These rate coefficients are consistent with our previous kinetic measurements at a higher acetylene number density where the rate coefficients of the individual reaction steps could not be determined, and an overall composite rate due to formation of the two products was measured as 4.2 ± 2.5 × 10^–11^ cm^3^ s^–1^ at 304 K.^20^ The measured rate coefficient of 2.1(±0.4) × 10^–11^ cm^3^ s^–1^ for the formation of the first benzonitrile-acetylene adduct (C_9_NH_7_^+•^, *m*/*z* 129) at 334.5 K is lower than the effective bimolecular association rate coefficient of 6.2(±0.3) × 10^–11^ cm^3^ s^–1^ but closer to the radiative rate coefficient of 3.8(±0.4) × 10^–11^ cm^3^ s^–1^, both measured at 150 K, as recently reported by Rap et al.^29^ However, our measured rate (1.1(±0.9) × 10^–11^ cm^3^ s^–1^) for the formation of the second adduct (C_11_NH_9_^+•^, *m*/*z* 155) is similar to the rate (1.3(±0.3) × 10^–11^ cm^3^ s^–1^) measured for the formation of two products (C_11_NH_8_^+^, *m*/*z* 154 and C_11_NH_9_^+•^, *m*/*z* 155) in the experiments of Rap et al.^29^ The similarity of the rate coefficients measured at the low number density of C_2_H_2_ (6 × 10^9^ – 3 × 10^11^) in the experiments by Rap et al. where termolecular association is unlikely,^29^ and the high number density (3.6 × 10^13^ – 1.0 × 10^14^) used in our experiments could suggest no or small contributions from termolecular reactions in our experiments. However, it should be noted that under our experimental conditions, the only two reaction products observed are the first and second acetylene adducts (C_9_NH_7_^+•^, *m*/*z* 129 and C_11_NH_9_^+•^, *m*/*z* 155, respectively), and the reaction channel leading to the formation of the H loss product (C_11_NH_8_^+^, *m*/*z* 154) as reported by Rap et al.,^29^ was not observed under our experimental conditions.

**Table 1:** Experimentally Determined Second Order Rate Coefficients for the Formation of the First (C_9_NH_7_^+•^) and Second (C_11_NH_9_^+•^) Addition Products of Acetylene on the Benzonitrile Radical Cation

*T* (°K)	*k*2 (cm^3^ s^–1^) (C_9_NH_7_^+•^)	*k*2 (cm^3^ s^–1^) (C_11_NH_9_^+•^)
334.5	2.1 (±0.4) × 10^–11^	1.1 (±0.9) × 10^–11^

The individual rate coefficients indicate reaction efficiencies (the ratio of the measured rate coefficient to the Langevin rate coefficient of 1.5 × 10^–9^ cm^3^ s^–1^) of 1.4% and 0.7% at 334.5 K for the formation of the first and second covalent adducts, respectively. These low reaction efficiencies for exothermic covalent bond forming reactions can be explained by considering the key feature of ion–molecule reactions which involve long-range attractive interactions between the ion and the neutral polarizable molecule.^3,31^ These attractive interactions often overcome reaction barriers, and therefore, most exothermic ion–molecule reactions involve no barriers and occur with high efficiencies approaching unit efficiency, thus occurring at the collision rate.^3,31^ However, it should be clear that not all exothermic ion–molecule reactions occur at the collision rate.^31^ Most commonly, reactions requiring significant rearrangements in intermediate complexes such as those resulting in covalent bond formation are often observed to occur at low efficiencies. For example, a wide range of reaction rates has been found for the sequential reactions of acetylene with ionized aromatics that covers very slow reactions involving large energy barriers as in the reactions of the benzene and styrene radical cations to very fast reactions occurring at the collision rate (100% reaction efficiency) as in the reaction of the pyrimidinium cation.^13,18,20^ The high reactivity of the phenylium ion (C_6_H_5_^+^) with acetylene resulting in the formation of the C_8_H_7_^+^ covalent ion with a 50% reaction efficiency is attributed to significantly reducing the barrier for the cation ring growth mechanism by the loss of a H atom from the benzene ring.^19,20^ The relatively low efficiencies (1.4% and 0.7%) measured for the reactions of acetylene with the benzonitrile radical cation suggest that the barrier to the cation ring growth mechanism still exists. However, these efficiencies are significantly higher than the efficiency of 0.001 measured for the reaction of acetylene with the phenylacetylene radical cation at 302 K.^20^ The current results therefore confirm the assumption that the cation ring growth mechanism can be significantly enhanced by the formation of N–C bonds.^18,20,29,32^

Ion mobility measurements can provide structural information on the gas phase ions through the measurement of the collision cross-section (Ω) of the ion with a buffer gas,^33,34^ which depends on the geometrical structure and shape of the ion. The most plausible ion structures are determined from geometry optimization calculations performed on several isomeric configurations, and angle-averaged collision cross sections (CCS) are computed for the lowest energy isomers using the Lennard-Jones scaled Projection Approximation (LJ-PA) method for the covalent structures^35^ and the Exact Hard Spheres (EHS) model for the noncovalent structures.^36^ The experimentally measured Ω’s are then compared to the theoretically calculated Ω’s to identify the most likely structures.

The mobility *K* of an ion is defined according to eq 1:^33,34^
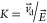
1where  is the drift velocity of the ion and E is the field across the drift region. The reduced mobility *K*_0_ (scaled to the number density at the standard temperature and pressure STP) is given by eq 2:
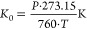
2where *P* is the pressure in Torr, and *T* is the temperature in Kelvin. Equations 1 and 2 can be combined and rearranged to give eq 3:
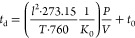
3where *l* is the drift distance (5 cm in our system), *t*_d_ is the mean arrival time of the drifting ion packet corrected for the non-Gaussian shape of the ATD peak,^33^*t*_0_ is the time that the ion spends outside the drift cell before it reaches the detector, and *V* is the voltage across the drift cell. The mobility measurements were carried out in the low-field limit defined as *E*/*N* < 5.0, where *E* is the electric field (V/cm) and *N* is the buffer gas density (cm^–3^) and *E*/*N* is typically expressed in units of Townsend (Td) where 1 Td = 10^–17^ V·cm^2^).^34^ The reduced mobility is determined according to eq 3, by plotting *t*_d_ versus *P/V*. The ATDs and the *t*_d_ versus *P/V* plots for the first (*m*/*z* 129) and second (*m*/*z* 155) acetylene adduct ions are displayed in Figure 3. The measurements yield reduced mobilities (*K*_0_) of 8.2 ± 0.3 cm^2^ V^–1^·s^–1^ and 7.5 ± 0.3 cm^2^ V^–1^·s^–1^ for the first and second adduct, respectively.

**Figure 3:**
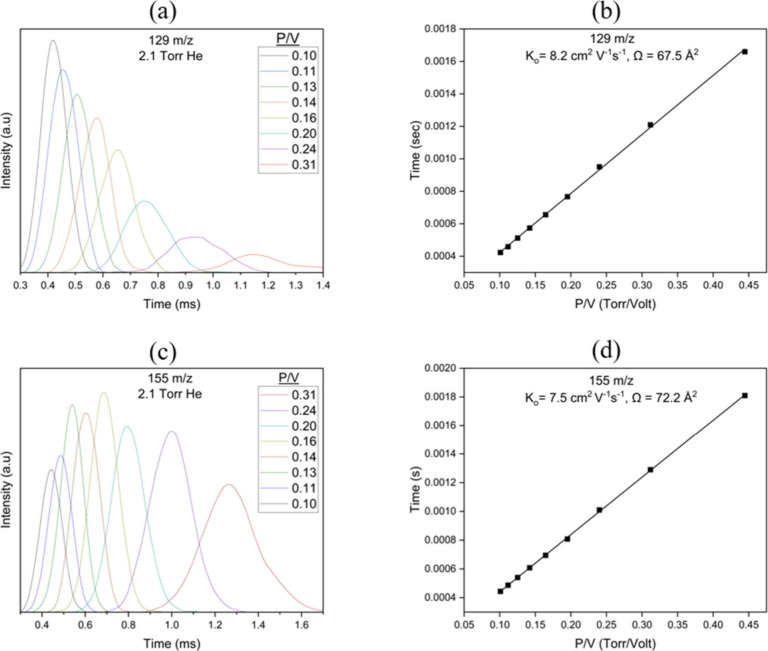
(a–d) ATDs of the benzonitrile-acetylene first (B^+•^ A, *m*/*z* 129) and second (B^+•^ A_2_, *m*/*z* 155) adducts at the drift cell conditions provided and the corresponding mobility plots.

The kinetic theory of gases^34^ relates the mobility of an ion to the average collision cross-section of the ion with the buffer gas according to eq 4:
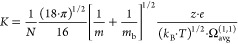
4where *N* is the number density of the buffer gas, *m* is the mass of the ion, *m*_b_ is the mass of a buffer gas atom, *z* is the number of charges, *e* is the electron charge, *k*_B_ is Boltzmann’s constant, and Ω_avg_^(1,1)^ is the average collision integral (collision cross-section). The measured reduced mobilities of the benzonitrile-acetylene first (*K*_0_ = 8.2 ± 0.3 cm^2^ V^–1^·s^–1^) and second (*K*_0_ = 7.5 ± 0.3 cm^2^ V^–1^·s^–1^) adducts result in Ω values of 67.5 Å^2^ and 72.2 Å^2^, respectively at 300 K. The significant increase (∼18%) in the CCS of the benzonitrile-acetylene adduct ion (C_9_NH_7_^+•^, Ω = 67.5 Å^2^) as compared to the benzonitrile radical cation (C_7_NH_5_^+•^, Ω = 57.4 Å^2^) is expected because of the addition of acetylene onto the C_7_NH_5_^+•^ ion. However, the second addition of acetylene on the C_9_NH_7_^+•^ ion results in only a small increase (∼7%) in the CCS of the C_11_NH_9_^+•^ ion (Ω = 72.2 Å^2^) as compared to the C_9_NH_7_^+•^ ion (Ω = 67.5 Å^2^). This suggests that the second addition of acetylene results in the formation of a less extended and a more compact structure of the C_11_NH_9_^+•^ ion as compared to the C_9_NH_7_^+•^ ion. The structures of the benzonitrile-acetylene adduct ions C_9_NH_7_^+•^ and C_11_NH_9_^+•^ are investigated and discussed below.

The observed benzonitrile-acetylene adduct ions C_9_NH_7_^+•^ and C_11_NH_9_^+•^ exhibit high thermal stability as evident by the lack of significant dissociation at temperatures as high as 593 K consistent with the formation of covalently bonded ions.^20^ In addition, the irreversible addition of acetylene on the benzonitrile radical cation is clearly established by the lack of equilibrium among the reactant and product ions, as demonstrated in the ATDs and reaction time profiles shown in Figure 2. Therefore, it can be concluded that the noncovalent structures of the benzonitrile-acetylene adduct ions are not observed under our experimental conditions. To further confirm this conclusion, we compare the measured CCS of the benzonitrile-acetylene adduct C_9_NH_7_^+•^ with the calculated CCSs for the lowest energy noncovalent cluster ions. Figure S4 (Supporting Information) shows the three lowest energy noncovalent structures of the benzonitrile-acetylene cluster ion with binding energies of 7.0, 6.8, and 4.6 kcal/mol. These structures are used to obtain average collision cross sections using the EHS model within the Mobcal program.^36^ The calculated CCSs corresponding to the noncovalent structures (72.1 Å^2^, 71.1 Å^2^, and 76.4 Å^2^) are about 7%, 5% and 13%, respectively higher than the measured Ω value at 300 K. This indicates that the noncovalent structures have almost no contributors to the measured collision cross-section of the benzonitrile-acetylene adduct.

Figure 4 presents the structures of the four lowest energy covalent ions (a), (b), (c), and (d) formed by the reaction of acetylene with the benzonitrile radical cation, with geometries optimized at the B97M-V/def2-SVPD level.^37−39^ The binding energies calculated using the CCSD(T) method at the complete basis set (CBS)^39^ limit reveal that the three lowest energy isomers (a), (b), and (c) are formed by the addition of acetylene on the para, ortho, and meta positions of the benzonitrile cation, respectively, and exhibit similar binding energies (within 1 kcal/mol). However, the formation of these structures requires 1,2 H-shifts following the addition of acetylene on the phenyl ring, which typically involve substantial energy barriers and therefore are not expected to form under the current experimental conditions involving thermalized and collisonally stabilized ions. Therefore, the N-acetylene-benzonitrile^+•^ radical cation (structure (d) in Figure 4) with a binding energy of 33.2 kcal/mol appears to be the most likely lowest energy covalent isomer that can be formed by the direct addition of acetylene on the benzonitrile radical cation. The structures of the second covalent adduct, C_11_NH_9_^+•^, were determined by identifying the lowest energy isomers formed by the addition of acetylene into isomer (d) in Figure 4. The structures of the three lowest energy isomers of the C_11_NH_9_^+•^ ion are shown in Figure 4(e), (f), and (g). Notably, the lowest energy isomer (g) corresponds to the bicyclic 2-phenylpyridine structure, which could explain the observed low reactivity toward the addition of the third acetylene molecule onto the benzonitrile cation after the formation of structure (g).

**Figure 4:**
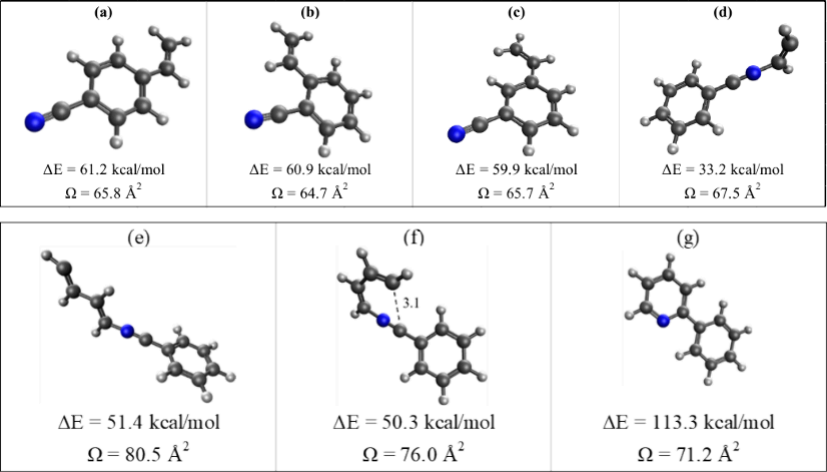
Most stable structures resulting from the first addition of acetylene to the benzonitrile radical cation (a–d). Structures (e) and (f) represent low energy isomers formed by the addition of acetylene to the covalent adduct (d), with structure (g) identified as the global minimum. All structures were optimized using the B97M-V/def2-SVPD level of theory, with energies calculated using CCSD(T)/CBS. Collision cross sections (Ω) were determined using the Projection Approximation^35^ based on the B97M-V/def2-SVPD optimized structures.

The structures of the covalent ions shown in Figure 4 are used to obtain average CCSs (Ω) using the Lennard-Jones scaled Projection Approximation (LJ-PA) method within the Sigma program.^35^ The calculated Ω values given in Figure 4 show that structure (d) has an Ω value of 67.5 Å^2^, which is in perfect agreement with the measured value of 67.5 Å^2^ (Figure 3(b)). Although the calculated CCSs (65–66 Å^2^) of structures (a), (b), and (c) are also in very good agreement with the measured Ω value, the formation of these structures can be excluded due to the substantial energy barriers required for the 1,2 H-shifts. Therefore, based on the ion mobility measurement, the observed benzonitrile-acetylene adduct (C_9_NH_7_^+•^) is most likely to have the structure of N-acetylene-benzonitrile^+•^ radical cation shown in Figure 4 (d).

The calculated Ω (71.2 Å^2^) of the lowest energy isomer of the C_11_NH_9_^+•^ ion (structure (g) shown in Figure 4) corresponding to the 2-phenylpyridine structure is in excellent agreement with the measured Ω of 72.2 Å^2^ (Figure 3(d)) for the second benzonitrile-acetylene adduct C_11_NH_9_^+•^. The formation of an extended open structure (isomer (e) in Figure 4) can be clearly excluded based on both the smaller binding energy (Δ*E* = 51.4 kcal/mol) and the larger CCS (Ω = 80.5 Å^2^) as compared to isomer (g) which has a significantly larger binding energy (Δ*E* = 113.3 kcal/mol) and a smaller CCS (Ω = 71.2 Å^2^) which compares very well with the measured Ω of 72.2 Å^2^. It is also interesting that isomer (f) in Figure 4 has a nearly similar binding energy (within 1 kcal/mol) to isomer (e) but a smaller CCS (Ω = 76.0 Å^2^) due to its more compact structure, which suggests that isomer (f) could represent an intermediate structure in the rearrangement of the extended open structure (e) to the bicyclic compact structure (g). We emphasize the value of calculating CCSs corresponding to different reaction intermediates leading to the formation of a compact cyclic structure and observing the systematic decrease in the CCSs as they converge to the smallest value corresponding to the lowest energy structure as clearly illustrated in the calculated CCSs of structures (e), (f), and (g) in Figure 4. Finally, the ion mobility assignment of the benzonitrile(acetylene)_2_ adduct (C_11_NH_9_^+•^) to the 2-phenylpyridine radical cation reported here is in full agreement with the IRMPD spectroscopic assignment of the C_11_NH_9_^+•^ product formed by the sequential reactions of acetylene with the benzonitrile radical cation at 150 K as reported recently by Rap et al.^29^ The confirmation of the structural identification of the 2-phenylpyridine radical cation by two independent measurements involving IR spectroscopy and CCSs under very different reaction conditions such as temperature (150–334 K), acetylene number density (6 × 10^9^ – 1 × 10^14^) and He number density (2 × 10^11^ – 7 × 10^16^) provides conclusive evidence for the formation of nitrogen-containing complex organics by the sequential reactions of acetylene with the benzonitrile radical cation. These results also provide strong justification for the search of N-acetylene-benzonitrile and 2-phenylpyridine in different regions of outer space.

It is important to compare our experimental and computational results with the potential energy surface (PES) calculations reported by Rap et al. for the sequential reactions of acetylene with the benzonitrile radical cation forming the observed 2-phenylpyridine product.^29^ Our results show that the binding energy of the N-acetylene-benzonitrile radical cation (structure (d) in Figure 4, 33.2 kcal/mol) is in excellent agreement with the binding energy of 32.1 kcal/mol calculated for the same structure shown in the PES of Rap et al. as the first covalent adduct of the acetylene-benzonitrile radical cation. However, while no infrared signature of the N-acetylene-benzonitrile structure was observed in the work of Rap et al., our measured CCS of the acetylene-benzonitrile radical cation (67.5 Å^2^) is in perfect agreement with the calculated CCS (67.5 Å^2^) of the N-acetylene-benzonitrile structure. Therefore, our results provide the first experimentally based structure of the acetylene-benzonitrile covalent adduct. It is also important to indicate that the noncovalent benzonitrile^+•^(acetylene) adducts were not observed under our experimental conditions where only the irreversible addition of acetylene onto the benzonitrile radical cation resulting in the formation of the covalent N-acetylene-benzonitrile^+•^ adduct (structure (d) in Figure 4) was observed based on the CCS measurement. As indicated above, the calculated CCSs of the noncovalent structures shown in Figure S4 (Supporting Information) are significantly larger than the measured CCS (67.5 Å^2^) of the benzonitrile^+•^(acetylene) adduct, which is in perfect agreement with the calculated CCS (67.5 Å^2^) of the N-acetylene-benzonitrile structure. This contrasts with the work of Rap et al. where a noncovalent prereactive complex was detected by infrared action spectroscopy, and a small barrier of only a few kJ/mol was calculated for the formation of the covalent N-acetylene-benzonitrile adduct.^29^ It is noted, however, that such a small barrier does not explain the low reaction efficiency of 1.4% or 2.5% measured for the formation of the covalent N-acetylene-benzonitrile adduct in both the current experiments or the experiments of Rap et al.,^29^ respectively.

Our computational results of the structure of the second benzonitrile-acetylene adduct (C_11_NH_9_^+•^) show three low energy structures ((e), (f), and (g) in Figure 4), but, only the lowest energy structure (g) shows an excellent agreement (Ω = 71.2 Å^2^) with the measured CCS (Ω = 72.2 Å^2^) of the C_11_NH_9_^+•^ ion. Clearly, the extended open structure ((e) in Figure 4) can be excluded based on both the smaller binding energy (Δ*E* = 51.4 kcal/mol) and the larger CCS (Ω = 80.5 Å^2^). The smaller binding energy of the prebicyclic structure (f) in Figure 4 compared to structure (e) in Figure 4 can serve as an upper limit estimate of the barrier (1.1 kcal/mol) required for the cyclization of the radical cation (e) in Figure 4 to form the 2-phenylpyridine (g) in Figure 4. In fact, this estimated barrier is in very good agreement with the barrier to ring closure (0.96 kcal/mol) calculated as the difference in energy between structures R6 and TSR4 in the PES of Rap et al.^26^

The mobility measurements indicate that the observed benzonitrile-acetylene adducts (C_9_NH_7_^+•^) and (C_11_NH_9_^+•^) are most likely to have the structures of N-acetylene-benzonitrile and 2-phenylpyridine, respectively (structures (d) and (g) in Figure 4). To explain the barrier for the ring closure of structure (d) shown in Figure 4, we propose that the addition of acetylene induces the rearrangement of structure (d) from the classical radical cation form (d1), shown in Scheme 1, to the distonic structure (e1) where the radical site is centered on the terminal carbon atom of the second acetylene molecule, and the charge site is centered at the carbon atom connected to the phenyl ring of the benzonitrile molecule as shown in Scheme 1 below.

**Scheme 1:**
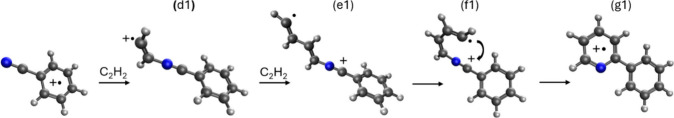


It should be noted that distonic structures involving substituted benzonitrile radical cations are known to form under different reaction conditions.^21,30,40,41^ However, there is usually a significant barrier to the classical → distonic rearrangement.^40,41^ On the other hand, the conversion of isomers (e) or (f) to isomer (g) is highly exothermic due to the stability of structure of the bicyclic structure of 2-phenylpyridine as indicated by the calculated binding energy shown in Figure 4 (Δ*E* = 113.3 kcal/mol). Therefore, it appears that the addition of the second acetylene molecule triggers the transition and conversion of the classical radical cation (d1) into the distonic structures in (e1) and (f1), which can efficiently cyclize in an exothermic transformation to form the classical radical cation 2-phenylpyridine (g1) shown in Scheme 1. In other words, the large exothermicity of the (f1) → (g1) rearrangement drives the transformation of the classical → distonic structures in (e1) and (f1) and results in an overall exothermic process generating the 2-phenylpyridine radical cation as structure (g) shown in Figure 4.

In conclusion, benzonitrile molecules are present in ionizing environments such as interstellar clouds and solar nebulae, where their ions can react with neutral molecules such as acetylene to form a variety of complex organics such as polycyclic aromatic hydrocarbons (PAHs) and polycyclic aromatic nitrogen heterocyclics (PANHs). The experimental and computational results reported here reveal the formation of two covalently bonded adduct ions C_9_NH_7_^+•^ and C_11_NH_9_^+•^ with binding energies of 33.2 and 113.3 kcal/mol, respectively, by the sequential reactions of acetylene with the benzonitrile radical cation. Kinetic measurements at 334.5 K determine the individual rate coefficients for the formation of the C_9_NH_7_^+•^ and C_11_NH_9_^+•^ ions as 2.1(±0.4) × 10^–11^ cm^3^ s^–1^ and 1.1(±0.9) × 10^–11^ cm^3^ s^–1^, respectively, thus demonstrating reaction efficiencies of 1.4% and 0.7%, respectively. Mass-selected ion mobility measurements reveal the formation of a N–C bond by the direct addition of acetylene onto the N atom of the benzonitrile cation, resulting in the formation of N-acetylene-benzonitrile with a calculated collision cross-section of 67.5 Å^2^ in perfect agreement with the measured cross-section of 67.5 Å^2^ of the C_9_NH_7_^+•^ adduct. The measured collision cross-section of the second covalent adduct C_11_NH_9_^+•^ (72.2 Å^2^) is also in excellent agreement with the calculated cross-section (71.2 Å^2^) of the lowest energy isomer of the C_11_NH_9_^+•^ ion corresponding to the 2-phenylpyridine structure. The formation of the bicyclic 2-phenylpyridine radical cation can be explained by the conversion of the classical radical cation C_11_NH_9_^+•^ into distonic structures that can efficiently cyclize in an exothermic transformation to form the radical cation of 2-phenylpyridine. This interesting kinetic behavior could represent a generalized reaction scheme that would have significant implications for the formation of complex organics in different regions of outer space. It would be interesting to explore whether similar structures, interactions, and mechanisms apply in the reactions of acetylene with larger N-containing ionized aromatics such as the 1-cyanonaphtalene and 2-cyanonathalene radical cations. These studies are currently under investigation in our laboratory, and we expect that the current along with the future results would have direct implications for the search for nitrogen-containing complex organics in space.

## Experimental and Computational Methods

The VCU mass-selected ion mobility spectrometer was used to perform the current experiments (Schematic is given in Figure S1, Supporting Information). The instrument has been described in several publications,^20,30,32^ and therefore, only a brief description of the experimental methods is given here. The instrument consists of four vacuum chambers housing the supersonic beam expansion system coupled to an electron impact (EI) ionization source, a quadrupole mass filter, a drift cell, and a second quadrupole mass spectrometer. Using the first quadrupole mass filter, the benzonitrile radical cations are mass-selected and then injected in 30–50 μs pulses into the drift cell containing an acetylene-helium gas mixture. The injection energies (13–15 eV, laboratory frame) used in the experiments are required to introduce the molecular ions against the outflow of helium/acetylene escaping through the drift cell orifice. The injected ions are thermalized by collisions with He or He/acetylene outside and inside the drift cell. At a cell pressure of 1.0 Torr at 300 K, about 10^4^ collisions of the injected ions with He or He/acetylene typically occur in less than 1 ms of residence time. Typically, about 10–100 collisions are usually needed for full ion thermalization, which can take place after the ions traverse only 1–2% of the drift cell.^17,18,20^ By scanning the second quadrupole mass filter coaxially positioned downstream from the drift cell, the reactant and product ions can be separated and analyzed. The ions are detected by an off-axis collision dynode and an electron multiplier after they exit the second quadrupole. The arrival time distributions (ATDs) of the selected ions exiting the cell are measured by a multichannel scalar triggered by the ion gate pulse. The actual partial pressure of acetylene in the drift cell was determined by measuring the rate of the reaction: C_2_H_2_^+•^ + C_2_H_2_ and using the previously reported rate constant for this reaction to obtain the number density of acetylene in the drift cell.^42^ The measured rate coefficients are typically reproduced three times, and errors were calculated based on the estimated uncertainties in acetylene partial pressure, temperature of the drift cell, fluctuations in the ion signal, and background noise.

All covalent and noncovalent radical cation structures were identified and optimized at the unrestricted B97M-V/def2-SVPD level of theory^37^,^38^ using a developed version of the Q-Chem software package.^39^ The binding interactions for acetylene with the benzonitrile cation (C_7_H_9_N) and with the covalent adduct C_9_H_11_N (Figure 4, parts a and d) were calculated at the CCSD(T)/CBS level using structures optimized at the B97M-V/def2-SVPD level. The CCSD(T)/CBS binding energies were calculated using a composite scheme that combines three terms: HF/aug-cc-pVQZ energies, MP2 correlation energies extrapolated to the CBS limit using aug-cc-pVTZ and aug-cc-pVQZ, and the ΔCCSD(T) correlation energies, which are determined from the difference between CCSD(T)/heavy-aug-cc-pVDZ and MP2/heavy-aug-cc-pVDZ correlation energies. All HF, MP2, and CCSD(T) calculations were performed using the restricted-open-shell formalism in the Psi4 software package^43^ to eliminate spin contamination in open-shell systems. The average collision cross sections (CCS) in helium were calculated for the lowest energy isomers using the Lennard-Jones scaled Projection Approximation (LJ-PA) method within the Sigma program for the covalent structures,^35^ and the Exact Hard Sphere (EHS) model within the Mobcal program for the noncovalent structures.^36^

## Supporting Information Available

Experimental setup of the mass-selected ion mobility system (Figure S1), mass spectra of the benzonitrile-acetylene adduct ions obtained at higher number density of acetylene (Figure S2), first-order kinetic plots for the reactions of C_7_NH_5_^+•^ and C_9_NH_7_^+•^ with acetylene (Figure S3), and noncovalent structures of the association of acetylene with the benzonitrile radical cation with their respective calculated binding energies and collision cross sections (Figure S4) (PDF)
Transparent Peer Review report available (PDF)
