# Supplementary material for: Sequential Reactions of Acetylene with the Benzonitrile Radical Cation: New Insights into Structures and Rate Coefficients of the Covalent Ion Products
Source: J Phys Chem Lett. 2024 Oct 29;15(44):11067–76. doi: 10.1021/acs.jpclett.4c02496 (PMC11552070; doi:10.1021/acs.jpclett.4c02496)
Supplement: Supplementary file 1 — jz4c02496_si_001.pdf [file jz4c02496_si_001.pdf]

Sequential Reactions of Acetylene with the Benzonitrile Radical Cation: New Insights into Structures and Rate Coefficients of the Covalent Ion Products

Paige Sutton, John Saunier, Ka Un Lao, and M. Samy El-Shall*

Department of Chemistry, Virginia Commonwealth University,
Richmond, VA 23284-2006, USA

Supporting Information

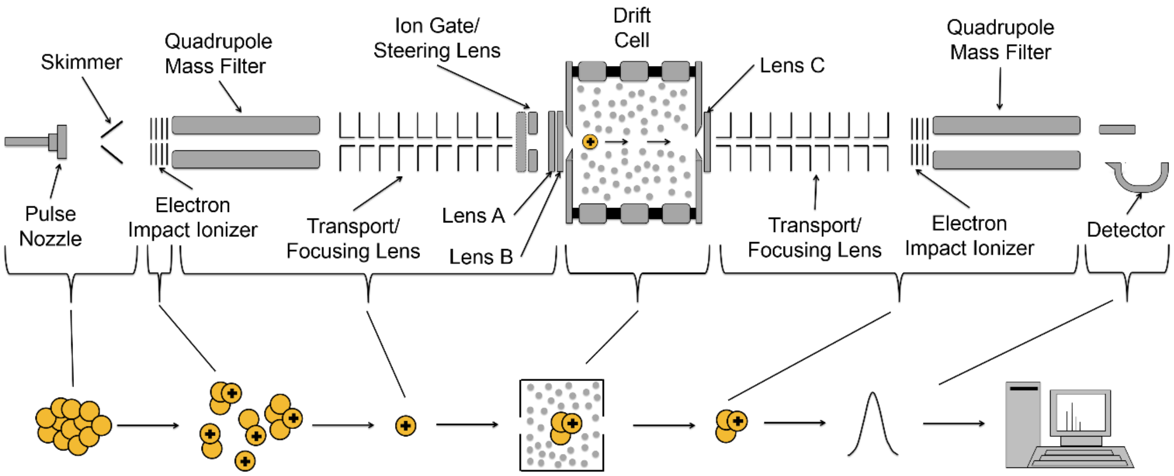

Figure S1. Experimental Set-up of the Mass-Selected Ion Mobility System.

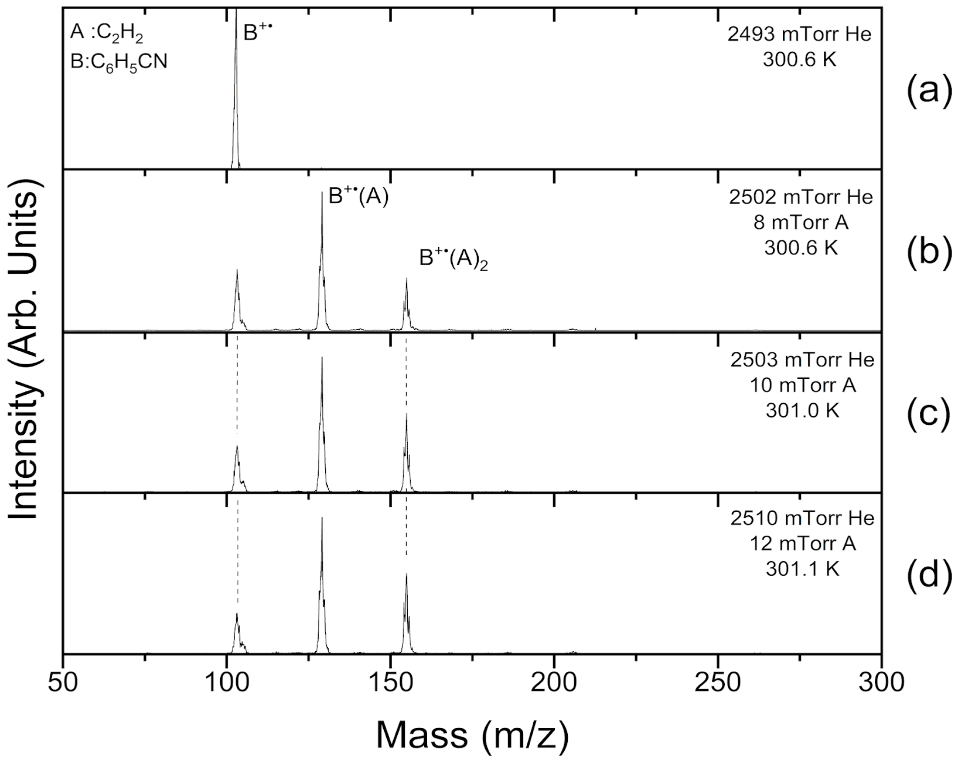

Figure S2. Mass spectra obtained from the injection (15 eV) of the benzonitrile radical cation ($B^{+\bullet}$, C_6H_5CN, *m/z* 103) into a drift cell containing: **(a)** 2.5 Torr of helium at 300.6 K and no acetylene (A, C_2H_2) and **(b-d)** with 8-12 mTorr acetylene at 301 K.

First-order kinetic plot corresponding to figure 2(c) for the formation of the first adduct.

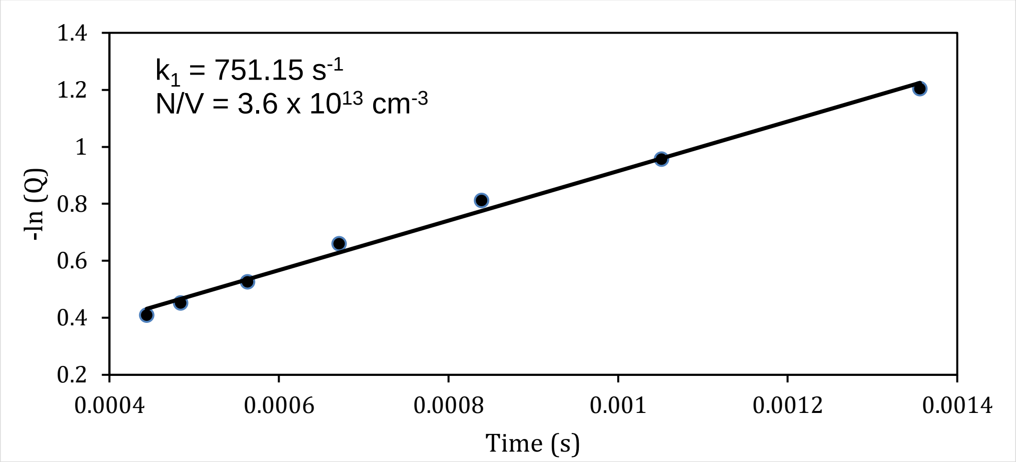

k1	751.15255
k2	2.098E-11

Pneut **(mTorr)**	1.2
T **(°C)**	61.3
Pneut **(atm)**	1.631E-06
T **(K)**	334.45
**# Density (prt cm-3)**	3.580E+13
k1 **(s-1)**	751.15255
k2 **(cm3 s-1 prt-1)**	2.098E-11

First-order kinetic plot corresponding to figure 2(d) for the formation of the second adduct.

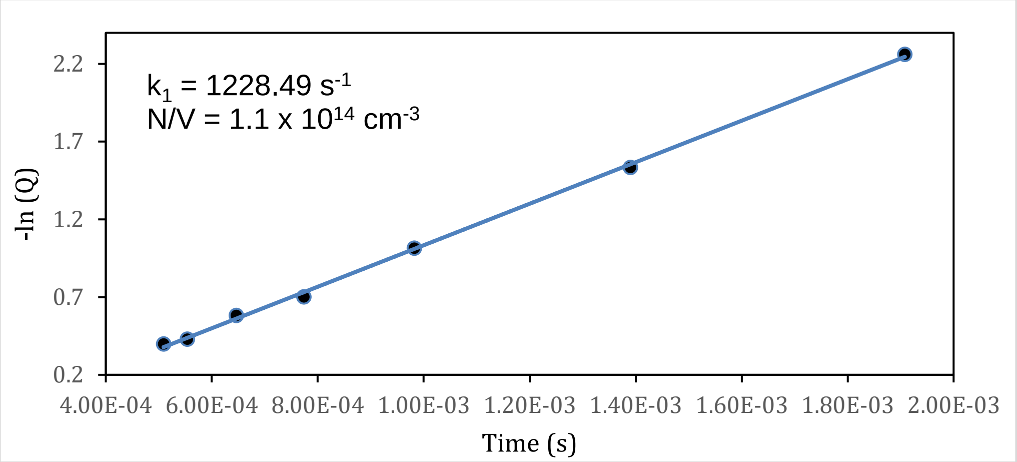

k1	1228.489716
k2	1.144E-11

Pneut **(mTorr)**	3.6
T **(°C)**	61.3
Pneut **(atm)**	4.89423E-06
T **(K)**	334.45
**# Density (prt cm-3)**	1.074E+14
k1 **(s-1)**	1228.489716
k2 **(cm3 s-1 prt-1)**	1.144E-11

Figure S3. First-order kinetic plots for the formation of the first and second Benzonitrile-Acetylene covalent adduct ions.

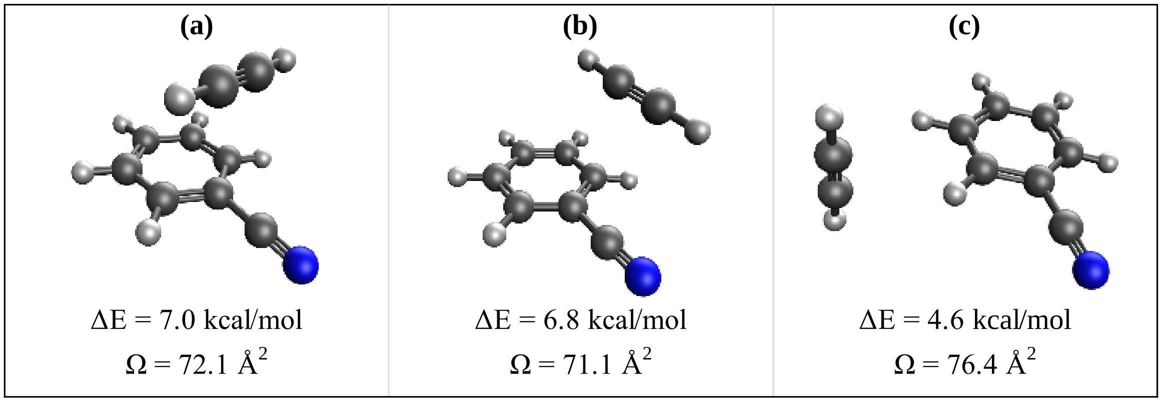

Figure S4. Noncovalent structures of the association of acetylene with the benzonitrile radical cation: **(a), (b)** and **(c)**, with their respective binding energies; structures optimized using B97M-V/def2-SVPD and energies calculated from RO-CCSD(T)/CBS. The collision cross-sections (Ω) were calculated from the Exact Hard Sphere (EHS) model of the Mobcal program[1] using the optimized structures at the B97M-V/def2-SVPD level of theory.

---

1. Shvartsburg, A. A.; Jarrold, M. F. An Exact Hard Spheres Scattering Model for the Mobilities of Polyatomic Ions, *Chem. Phys. Letters* **1996**, 261, 86-91.
