# Supplementary material for: Sequential Reactions of Acetylene with the Benzonitrile Radical Cation: New Insights into Structures and Rate Coefficients of the Covalent Ion Products
Source: J Phys Chem Lett. 2024 Oct 29;15(44):11067–76. doi: 10.1021/acs.jpclett.4c02496 (PMC11552070; doi:10.1021/acs.jpclett.4c02496)
Supplement: Supplementary file 2 — jz4c02496_si_002.pdf [file jz4c02496_si_002.pdf]

jz-2024-02496h.R1

Name: Peer Review Information for "Sequential Reactions of Acetylene with the Benzonitrile Radical Cation: New Insights into Structures and Rate Coefficients of the Covalent Ion Products"

First Round of Reviewer Comments

Reviewer: 1

Comments to the Author

This paper by Sutton et al. examines the reaction of the benzonitrile radical cation with acetylene in the gas phase – reactions are performed in a drift cell which is situated between two quadrupole mass filters. The key point of the paper, on my reading, is the formation of bicyclic product species from the addition of two acetylene molecules in succession. Some verification of this product assignment comes from CCS measurements – aided also by some calculations of relative stability of likely isomers. Second order rate coefficients for each reaction step with acetylene are reported.

It's an interesting result – and shows how radical ions can proceed in molecular weight growth. And these experiments are related to previous reports from lower temperature ion trap studies (at 150K). Rap et al. have already reported the spectroscopy of product of this reaction – so this paper verifies that result (albeit under difference physical conditions). Since these products have already been reported from this reaction – Im not sure there's sufficient impact for JPC Lett. It likely would make a good JPCA paper.

May main concerns are:

1. These same products have been spectroscopically assigned by Rap et al. from this same reaction so it is unclear what new insights these CCS result bring.

2. Rap et al also report detailed energy surface calculations for both reaction steps – how does SCHEME 1 compare to the mechanism in those calculations?

3. These rate coefficients are measured at 2.7 Torr and compared to the experimental work of Rap et al. there is about 6 to 7 orders of magnitude difference in the pressure. The Rap et al. measurement is undoubtedly under the low-pressure limit – but at 2.7 Torr, im not so sure. Have the authors considered that these reactions may be affected by the buffer gas – since there are collisions every ca. 10 ns? Im wondering if these reactions are within the “fall off” regime where the bimolecular rate will be affected by termolecular reactions and thus are pressure sensitive. These rates might only be relevant for this specific pressure (and temperature). Some discussion is needed here and also more careful text should be added to the text when comparing to the very low pressure work of Rap et al.

4. Again, the energy surface appears in Rap et al. so these rather low reaction efficiencies need to be explained – considering the large exothermicity of the addition reaction step … why are these reactions only ca 1% efficient?

5. Also on the kinetic measurements, are the kinetic rates extracted from traces like shown in Figure 2? It would be valuable to see a representative kinetic trace fitted with the appropriate exponential function and some representation of the quality of that fit. That could be added to a Supp Info section. Have pseudo first order conditions with respect to acetylene been verified?

Reviewer: 2

Comments to the Author

The manuscript presents a combined experimental and theoretical study on the sequential addition of two acetylene molecules to benzonitrile radical cation eventually leading to the formation of 2-phenylpyridine radical cation representing the lowest energy isomer of the $C_{11}NH_9^{+\bullet}$ ion. The authors used state-of-the-art mass spectrometry and ion-mobility techniques to identify the reaction products by their collision cross sections and to measure thermal rate constants for the first and second reactions. The significance of this work is that it provides a laboratory insight into the growth of polycyclic N-containing aromatic compounds (PNAH) in astrochemical environments. Interestingly, the presence of a 'bare' N atom seems to facilitate the ion-neutral reaction which is on the contrary to the growth of polycyclic aromatics in radical-neutral reactions.

The theoretical methods used to determine the geometries of various product isomers and the reaction energies are adequate and should be sufficiently accurate. However, there is a glaring omission in the theoretical part, which does not allow me to recommend this manuscript for publication without major revision. The authors did not consider transition states and reaction

barriers along the suggested reaction pathways. Without their inclusion, the proposed reaction mechanism remains largely speculative as kinetic control in the studied system is more likely than the thermodynamic control upon which the authors rely in the present version of the manuscript. The identification of transition states and reaction barriers would allow the authors to also theoretically evaluate the reaction rate constants, to compare them with the experimental results and to extrapolate the measured values to different temperatures of interest.

Author's Response to Peer Review Comments:

Please see attached the list of changes made in response to the Reviewers' comments.

List of Changes made in Response to Reviewers' Comments of Manuscript (jz-2024-02496h)

"*Sequential Reactions of Acetylene with the Benzonitrile Radical Cation: New Insights into Structures and Rate Coefficients of the Covalent Ion Products*"

Reviewer: 1

May main concerns are:

1. "These same products have been spectroscopically assigned by Rap et al. from this same reaction so it is unclear what new insights these CCS result bring."

Our CCS results provide the first experimental-based assignment of the structure of the first covalent adduct namely, the N-acetylene-benzonitrile radical cation. Our results show that the binding energy of the N-acetylene-benzonitrile radical cation is 33.2 kcal/mol in excellent agreement with the binding energy of 32.1 kcal/mol for the same structure shown in the PES of Rap *et. al.* However, while no infrared signature of the N-acetylene-benzonitrile structure was observed in the work of Rap *et al*., our measured CCS of the acetylene-benzonitrile radical cation (67.5 Å^2) is in perfect agreement with the calculated CCS (67.5 Å^2) of the N-acetylene-benzonitrile structure. This information in now included on Page 13 of the revised manuscript.

2. "Rap et al also report detailed energy surface calculations for both reaction steps – how does SCHEME 1 compare to the mechanism in those calculations?"

We now provide a detailed comparison of our experimental and computational results with the PES calculations reported by Rap *et. al.* on pages 13-14 of the revised manuscript. As discussed on Page 14, our upper limit estimate of the barrier (1.1 kcal/mol) required for the cyclization of the radical cation ((e) in Fig. 4) to form the 2phenylpyridine ((g) in Fig. 4), is in very good agreement with the barrier to ring closure (0.96 kcal/mol) calculated in the PES of Rap *et. al.*

3. "These rate coefficients are measured at 2.7 Torr and compared to the experimental work of Rap et al. there is about 6 to 7 orders of magnitude difference in the pressure. The Rap et al.

measurement is undoubtedly under the low-pressure limit – but at 2.7 Torr, im not so sure. Have the authors considered that these reactions may be affected by the buffer gas – since there are collisions every ca. 10 ns? Im wondering if these reactions are within the "fall off" regime where the bimolecular rate will be affected by termolecular reactions and thus are pressure sensitive. These rates might only be relevant for this specific pressure (and temperature). Some discussion is needed here and also more careful text should be added to the text when comparing to the very low pressure work of Rap et al."

As discussed on Page 8 of the revised manuscript, the similarity of the rate coefficients measured at the low number density of C_2H_2 ($6x10^9$ – $3x10^{11}$) in the experiments by Rap *et. al*. where termolecular association is unlikely, and the high number density ($3.6x10^{13}$ – $1.0x10^{14}$) used in our experiments could suggest no or small contributions from termolecular reactions in our experiments.

As discussed on Page 12 of the revised manuscript, the confirmation of the structural identification of the 2-phenylpyridine radical cation by two independent measurements involving IR spectroscopy and CCSs under very different reaction conditions such as temperature (150 – 334 K), acetylene number density ($6x10^9$ – $1x10^{14}$) and He number density ($2x10^{11}$ – $7x10^{16}$) provides occlusive evidence for the formation of nitrogencontaining complex organics by the sequential reactions of acetylene with the benzonitrile radical cation. These results also provide strong justification for the search of N-acetylene-benzonitrile and 2-phenylpyridine in different regions of outer space.

4. "Again, the energy surface appears in Rap et al. so these rather low reaction efficiencies need to be explained – considering the large exothermicity of the addition reaction step … why are these reactions only ca 1% efficient?"

The low reaction efficiencies for exothermic covalent bond forming reactions is now discussed in detail on Page 8 of the revised manuscript. Ion-molecule reactions involve long-range attractive interactions between the ion and the neutral molecule which often overcome reaction barriers and therefore, exothermic ion-molecule reactions are often observed to be barrierless and to occur with high efficiencies. However, not all exothermic ion-molecule reactions occur efficiently. Most commonly, reactions requiring significant rearrangements in intermediate complexes as those resulting in covalent bond formation are often observed to occur at low efficiencies. For example, a wide range of reaction rates has been found for the sequential reactions of acetylene with ionized aromatics ranging from very slow reactions with large energy barriers as in the case of benzene and styrene radical cations to reactions occurring at the collision rate (100% reaction efficiency) as in the case of the pyrimidinium cation.[13,18,20] The relatively low efficiencies (1.4% and 0.7%) measured for the acetylene reactions with the benzonitrile radical cation suggest that the barrier to the cation ring growth mechanism still exists.

5. "Also on the kinetic measurements, are the kinetic rates extracted from traces like shown in Figure 2? It would be valuable to see a representative kinetic trace fitted with the appropriate exponential function and some representation of the quality of that fit. That could be added to a Supp Info section. Have pseudo first order conditions with respect to acetylene been verified?"

As discussed on Page 6 of the revised manuscript, pseudo first-order rate constants are calculated from ln $I/\Sigma I = -kt$, where I is the integrated intensity of the ATD peak of the reactant ion ($B^{+\bullet}$, $C_7NH_5^{+\bullet}$), and ΣI is sum of the integrated intensities of the reactant and the product ion peaks ($B^{+\bullet}$ + $B^{+\bullet}A$), obtained from the areas of their ATD peaks, and t is the mean drift time taken as the center of the ATD peak of the reactant ion.[20] The pseudo-first-order rate coefficients k_1 are obtained from the slopes of the plots of ln $I/\Sigma I$ vs t as shown in **Figure S3** (Supporting Information). The second-order rate constants k_2 are then obtained from the equation $k_2 = k_1/[N]$, where N is the number density of the acetylene partial pressure in the drift cell (3×10^{13} - 1×10^{14} cm^{-3}).

Reviewer: 2

Comments:
"The theoretical methods used to determine the geometries of various product isomers and the reaction energies are adequate and should be sufficiently accurate. However, there is a glaring omission in the theoretical part, which does not allow me to recommend this manuscript for publication without major revision. The authors did not consider transition states and reaction barriers along the suggested reaction pathways. Without their inclusion, the proposed reaction mechanism remains largely speculative as kinetic control in the studied system is more likely than the thermodynamic control upon which the authors rely in the present version of the manuscript. The identification of transition states and reaction barriers would allow the authors to also theoretically evaluate the reaction rate constants, to compare them with the experimental results and to extrapolate the measured values to different temperatures of interest."

As discussed on Pages 13-14 of the revised manuscript, Rap *et. al.* (Ref. 29) reported detailed potential energy surface (PES) calculations for the sequential reactions of acetylene with the benzonitrile radical cation forming the observed 2-phenylpyridine product. Our experimental and computational results are consistent with the PES calculations of Rap *et. al.* For example, our calculated binding energy of the first covalent adduct N-acetylene-benzonitrile radical cation (33.2 kcal/mol) is in excellent agreement with the binding energy of 32.1 kcal/mol for the same structure shown in the PES of Rap *et. al.* However, while no infrared signature of the N-acetylenebenzonitrile structure was observed in the work of Rap *et. al.*, our measured CCS of the acetylene-benzonitrile radical cation (67.5 Å^2) is in perfect agreement with the calculated CCS (67.5 Å^2) of the N-acetylene-benzonitrile structure. Therefore, our results provide the first experimental-based structure of the acetylene-benzonitrile covalent adduct.

Also, our upper limit estimate of the barrier (1.1 kcal/mol) required for the cyclization of the radical cation ((e) in Fig. 4) to form the 2-phenylpyridine ((g) in Fig. 4), is in very good agreement with the barrier to ring closure (0.96 kcal/mol) calculated in the PES of Rap *et. al.*

Second Round of Reviewer Comments

Reviewer: 2

Comments to the Author

I am satisfied with the authors responses and recommend publication of this manuscript in its present form.

Reviewer: 1

Comments to the Author

The authors have done a good job addressing the comments of the reviewers. The manuscript is improved and publishable.

As a final comment, I think its important to note that the reactivity of this ion molecular reaction is still not fully explained ... the Rap et al. paper for this reaction reports a pre-reactive complex with a forward barrier of only a few kJ/mol forward. As such, Im not convince this explains only ca. 1% forward reaction efficiency as this small forward barrier doesnt explain ca. 99% re-dissociation to the reactants. So either than barrier isnt correctly calculated - or there is an issue with the mechanism. In any case, my comment does not hold up the publication of this manuscript - but it is something the authors might want to consider (perhaps the rate constant for C2D2 in a future study?).

Author's Response to Peer Review Comments:

We have made all the suggested changes noted by Reviewer 1 (Pages 13-14 of the revised manuscript). We also revised the writing of the paper to eliminate any significant overlap with our published work and made all the formatting changes requested by the Editorial Office. With all the changes made, we believe the manuscript is now appropriate for publication in the Journal of Physical Chemistry Letters.

<u>List of Changes made in Response to Reviewers' Comments of Manuscript (jz-2024-02496h)</u>

"Sequential Reactions of Acetylene with the Benzonitrile Radical Cation: New Insights into Structures and Rate Coefficients of the Covalent Ion Products"

Reviewer: 1

May main concerns are:

1. “These same products have been spectroscopically assigned by Rap et al. from this same reaction so it is unclear what new insights these CCS result bring.”

Our CCS results provide the first experimental-based assignment of the structure of the first covalent adduct namely, the N-acetylene-benzonitrile radical cation. Our results show that the binding energy of the N-acetylene-benzonitrile radical cation is 33.2 kcal/mol in excellent agreement with the binding energy of 32.1 kcal/mol for the same structure shown in the PES of Rap *et. al.* However, while no infrared signature of the N-acetylene-benzonitrile structure was observed in the work of Rap *et al*., our measured CCS of the acetylene-benzonitrile radical cation (67.5 $Å^2$) is in perfect agreement with the calculated CCS (67.5 $Å^2$) of the N-acetylene-benzonitrile structure. This information in now included on Page 13 of the revised manuscript.

2. “Rap et al also report detailed energy surface calculations for both reaction steps – how does SCHEME 1 compare to the mechanism in those calculations?”

We now provide a detailed comparison of our experimental and computational results with the PES calculations reported by Rap *et. al.* on pages 13-14 of the revised manuscript. As discussed on Page 14, our upper limit estimate of the barrier (1.1 kcal/mol) required for the cyclization of the radical cation ((e) in Fig. 4) to form the 2phenylpyridine ((g) in Fig. 4), is in very good agreement with the barrier to ring closure (0.96 kcal/mol) calculated in the PES of Rap *et. al.*

3. “These rate coefficients are measured at 2.7 Torr and compared to the experimental work of Rap et al. there is about 6 to 7 orders of magnitude difference in the pressure. The Rap et al. measurement is undoubtedly under the low-pressure limit – but at 2.7 Torr, im not so sure. Have the authors considered that these reactions may be affected by the buffer gas – since there are collisions every ca. 10 ns? Im wondering if these reactions are within the “fall off” regime where the bimolecular rate will be affected by termolecular reactions and thus are pressure sensitive. These rates might only be relevant for this specific pressure (and temperature). Some discussion is needed here and also more careful text should be added to the text when comparing to the very low pressure work of Rap et al.”

As discussed on Page 8 of the revised manuscript, the similarity of the rate coefficients measured at the low number density of C_2H_2 ($6x10^9$ – $3x10^{11}$) in the experiments by Rap *et. al*. where termolecular association is unlikely, and the high number density ($3.6x10^{13}$ – $1.0x10^{14}$) used in our experiments could suggest no or small contributions from termolecular reactions in our experiments.

As discussed on Page 12 of the revised manuscript, the confirmation of the structural identification of the 2-phenylpyridine radical cation by two independent measurements involving IR spectroscopy and CCSs under very different reaction conditions such as temperature (150 – 334 K), acetylene number density ($6x10^9$ – $1x10^{14}$) and He number density ($2x10^{11}$ – $7x10^{16}$) provides occlusive evidence for the formation of nitrogencontaining

complex organics by the sequential reactions of acetylene with the benzonitrile radical cation. These results also provide strong justification for the search of N-acetylene-benzonitrile and 2-phenylpyridine in different regions of outer space.

4. "Again, the energy surface appears in Rap et al. so these rather low reaction efficiencies need to be explained – considering the large exothermicity of the addition reaction step … why are these reactions only ca 1% efficient?"

The low reaction efficiencies for exothermic covalent bond forming reactions is now discussed in detail on Page 8 of the revised manuscript. Ion-molecule reactions involve long-range attractive interactions between the ion and the neutral molecule which often overcome reaction barriers and therefore, exothermic ion-molecule reactions are often observed to be barrierless and to occur with high efficiencies. However, not all exothermic ion-molecule reactions occur efficiently. Most commonly, reactions requiring significant rearrangements in intermediate complexes as those resulting in covalent bond formation are often observed to occur at low efficiencies. For example, a wide range of reaction rates has been found for the sequential reactions of acetylene with ionized aromatics ranging from very slow reactions with large energy barriers as in the case of benzene and styrene radical cations to reactions occurring at the collision rate (100% reaction efficiency) as in the case of the pyrimidinium cation.[13,18,20] The relatively low efficiencies (1.4% and 0.7%) measured for the acetylene reactions with the benzonitrile radical cation suggest that the barrier to the cation ring growth mechanism still exists.

5. "Also on the kinetic measurements, are the kinetic rates extracted from traces like shown in Figure 2? It would be valuable to see a representative kinetic trace fitted with the appropriate exponential function and some representation of the quality of that fit. That could be added to a Supp Info section. Have pseudo first order conditions with respect to acetylene been verified?"

As discussed on Page 6 of the revised manuscript, pseudo first-order rate constants are calculated from $\ln I/\Sigma I = -kt$, where I is the integrated intensity of the ATD peak of the reactant ion ($B^{+\bullet}$, $C_7NH_5^{+\bullet}$), and ΣI is sum of the integrated intensities of the reactant and the product ion peaks ($B^{+\bullet} + B^{+\bullet}A$), obtained from the areas of their ATD peaks, and t is the mean drift time taken as the center of the ATD peak of the reactant ion.[20] The pseudo-first-order rate coefficients k_1 are obtained from the slopes of the plots of $\ln I/\Sigma I$ vs t as shown in **Figure S3** (Supporting Information). The second-order rate constants k_2 are then obtained from the equation $k_2 = k_1/[N]$, where N is the number density of the acetylene partial pressure in the drift cell (3×10^{13} - 1×10^{14} cm^{-3}).

Reviewer: 2

Comments:
"The theoretical methods used to determine the geometries of various product isomers and the reaction energies are adequate and should be sufficiently accurate. However, there is a glaring omission in the theoretical part, which does not allow me to recommend this manuscript for publication without major revision. The authors did not consider transition states and reaction barriers along the suggested reaction pathways. Without their inclusion, the proposed reaction mechanism remains largely speculative as kinetic control in the studied system is more likely than the thermodynamic control upon which the authors rely in the present version of the

manuscript. The identification of transition states and reaction barriers would allow the authors to also theoretically evaluate the reaction rate constants, to compare them with the experimental results and to extrapolate the measured values to different temperatures of interest."

As discussed on Pages 13-14 of the revised manuscript, Rap *et. al.* (Ref. 29) reported detailed potential energy surface (PES) calculations for the sequential reactions of acetylene with the benzonitrile radical cation forming the observed 2-phenylpyridine product. Our experimental and computational results are consistent with the PES calculations of Rap *et. al.* For example, our calculated binding energy of the first covalent adduct N-acetylene-benzonitrile radical cation (33.2 kcal/mol) is in excellent agreement with the binding energy of 32.1 kcal/mol for the same structure shown in the PES of Rap *et. al.* However, while no infrared signature of the N-acetylenebenzonitrile structure was observed in the work of Rap *et. al.*, our measured CCS of the acetylene-benzonitrile radical cation (67.5 Å^2) is in perfect agreement with the calculated CCS (67.5 Å^2) of the N-acetylene-benzonitrile structure. Therefore, our results provide the first experimental-based structure of the acetylene-benzonitrile covalent adduct.

Also, our upper limit estimate of the barrier (1.1 kcal/mol) required for the cyclization of the radical cation ((e) in Fig. 4) to form the 2-phenylpyridine ((g) in Fig. 4), is in very good agreement with the barrier to ring closure (0.96 kcal/mol) calculated in the PES of Rap *et. al.*
